# Effects of high altitude on respiratory rate and oxygen saturation reference values in healthy infants and children younger than 2 years in four countries: a cross-sectional study

**DOI:** 10.1016/S2214-109X(19)30543-1

**Published:** 2020-02-19

**Authors:** Mary E Crocker, Shakir Hossen, Dina Goodman, Suzanne M Simkovich, Miles Kirby, Lisa M Thompson, Ghislaine Rosa, Sarada S Garg, Gurusamy Thangavel, Eric D McCollum, Jennifer Peel, Thomas Clasen, William Checkley, HAPIN Investigators

**Affiliations:** a Department of Paediatrics, School of Medicine, University of Washington, Seattle, WA, USA; b Division of Pulmonary and Sleep Medicine, Seattle Children's Hospital, Seattle, WA, USA; c Division of Pulmonary and Critical Care, Johns Hopkins University School of Medicine, Baltimore, MD, USA; d Centre for Global Non-Communicable Disease Research and Training, Johns Hopkins University School of Medicine, Baltimore, MD, USA; e Eudowood Division of Paediatric Respiratory Sciences, Department of Paediatrics, Johns Hopkins University School of Medicine, Baltimore, MD, USA; f Department of International Health, Bloomberg School of Public Health, Johns Hopkins University School of Medicine, Baltimore, MD, USA; g Department of Environmental Health, Rollins School of Public Health, Emory University, Atlanta, GA, USA; h Nell Hodgson Woodruff School of Nursing, Emory University, Atlanta, GA, USA; i Department of Disease Control, London School of Hygiene and Tropical Medicine, London, UK; j Department of Environmental Health Engineering, ICMR Centre for Advanced Research on Air Quality, Climate and Health, Sri Ramachandra Institute of Higher Education and Research (SRIHER), Chennai, India; k Department of Environmental and Radiological Health Sciences, Colorado State University, Fort Collins, CO, USA

## Abstract

**Background:**

In resource-limited settings, pneumonia diagnosis and management are based on thresholds for respiratory rate (RR) and oxyhaemoglobin saturation (SpO_2_) recommended by WHO. However, as RR increases and SpO_2_ decreases with elevation, these thresholds might not be applicable at all altitudes. We sought to determine upper thresholds for RR and lower thresholds for SpO_2_ by age and altitude at four sites, with altitudes ranging from sea level to 4348 m.

**Methods:**

In this cross-sectional study, we enrolled healthy children aged 0–23 months who lived within the study areas in India, Guatemala, Rwanda, and Peru. Participants were excluded if they had been born prematurely (<37 weeks gestation); had a congenital heart defect; had history in the past 2 weeks of overnight admission to a health facility, diagnosis of pneumonia, antibiotic use, or respiratory or gastrointestinal signs; history in the past 24 h of difficulty breathing, fast breathing, runny nose, or nasal congestion; and current runny nose, nasal congestion, fever, chest indrawing, or cyanosis. We measured RR either automatically with the Masimo Rad-97, manually, or both, and measured SpO_2_ with the Rad-97. Trained staff measured RR in duplicate and SpO_2_ in triplicate in children who had no respiratory symptoms or signs in the past 2 weeks. We estimated smooth percentiles for RR and SpO_2_ that varied by age and site using generalised additive models for location, shape, and scale. We compared these data with WHO RR and SpO_2_ thresholds for tachypnoea and hypoxaemia to determine agreement.

**Findings:**

Between Nov 24, 2017, and Oct 10, 2018, we screened 2027 children for eligibility. 335 were ineligible, leaving 1692 eligible participants. 30 children were excluded because of missing values and 92 were excluded because of measurement or data entry errors, leaving 1570 children in the final analysis. 404 participants were from India (altitude 1–919 m), 389 were from Guatemala (1036–2017 m), 341 from Rwanda (1449–1644 m), and 436 from Peru (3827–4348 m). Mean age was 7·2 months (SD 7·2) and 796 (50·7%) of 1570 participants were female. Although average age was mostly similar between settings, the average participant age in Rwanda was noticeably younger, at 5·5 months (5·9). In the 1570 children included in the analysis, mean RR was 31·9 breaths per min (SD 7·1) in India, 41·5 breaths per min in Guatemala (8·4), 44·0 breaths per min in Rwanda (10·8), and 48·0 breaths per min in Peru (9·4). Mean SpO_2_ was 98·3% in India (SD 1·5), 97·3% in Guatemala (2·4), 96·2% in Rwanda (2·6), and 89·7% in Peru (3·5). Compared to India, mean RR was 9·6 breaths per min higher in Guatemala, 12·1 breaths per min higher in Rwanda, and 16·1 breaths per min higher in Peru (likelihood ratio test p<0·0001). Smooth percentiles for RR and SpO_2_ varied by site and age. When we compared age-specific and site-specific 95th percentiles for RR and 5th percentiles for SpO_2_ against the WHO cutoffs, we found that the proportion of false positives for tachypnoea increased with altitude: 0% in India (95% CI 0–0), 7·3% in Guatemala (4·1–10·4), 16·8% in Rwanda (12·9–21·1), and 28·9% in Peru (23·7–33·0). We also found a high proportion of false positives for hypoxaemia in Peru (11·6%, 95% CI 7·0–14·7).

**Interpretation:**

WHO cutoffs for fast breathing and hypoxaemia overlap with RR and SpO_2_ values that are normal for children in different altitudes. Use of WHO definitions for fast breathing could result in misclassification of pneumonia in many children who live at moderate to high altitudes and show acute respiratory signs. The 5th percentile for SpO_2_ was in reasonable agreement with the WHO definition of hypoxaemia in all regions except for Peru (the highest altitude site). Misclassifications could result in inappropriate management of paediatric respiratory illness and misdirection of potentially scarce resources such as antibiotics and supplemental oxygen. Future studies at various altitudes are needed to validate our findings and recommend a revision to current guidelines. Substantiating research in sick children is still needed.

**Funding:**

US National Institutes of Health, Bill & Melinda Gates Foundation.

**Research in context**

**Evidence before this study**

We searched PubMed with various combinations of the terms “respiratory rate”, “oxygen saturation” or “oxyhaemoglobin saturation”, “children”, “neonates”, and “altitude” for articles in English published at any point before June 26, 2019. Previous studies have shown that respiratory rate is higher and oxyhaemoglobin saturation is lower in children living at high altitudes compared to those living at lower altitudes; however, earlier studies have typically included only one or two settings and used different methods from one another, making comparisons across studies difficult. Moreover, the commonly used WHO guidelines for pneumonia diagnosis and management were developed using data from studies at altitudes lower than 1600 m. Consequently, these guidelines might not be directly applicable to children living at higher altitudes.

**Added value of this study**

We compared respiratory rate and oxyhaemoglobin saturation of healthy children younger than 2 years at altitudes of four sites ranging from sea level to 4348 m and using consistent protocols and methods. As expected, respiratory rate was higher and oxyhaemoglobin saturation was lower with higher altitudes. As a result, application of the WHO guidelines would lead to misclassifying a large proportion of children living at high altitudes who present to a health facility with respiratory signs as having tachypnoea or hypoxaemia.

**Implications of all the available evidence**

Our study quantifies the extent of potential misclassification that results from use of the WHO guidelines, which do not adequately account for altitude. These results call for a re-examination of the definitions of tachypnoea and hypoxaemia used in global pneumonia management guidelines and suggest that development of new thresholds that better account for altitude could lead to improved case management and more appropriate use of scarce resources.

## Introduction

Pneumonia is a leading cause of death in children younger than 5 years, resulting in 700 000–900 000 deaths per year.^1^, ^2^ According to WHO, 15 countries account for 70% of the worldwide childhood pneumonia mortality.^3^ Ten of these countries include people living at elevations over 1500 m above sea level, and five (India, Pakistan, Ethiopia, China, and Afghanistan) have people living at elevations over 3000 m. In many resource-limited countries located at high altitude, the diagnosis and management of childhood pneumonia relies on thresholds for respiratory rate (RR) and oxyhaemoglobin saturation (SpO_2_) recommended by WHO.^4^, ^5^ However, local populations in these locations also tend to have higher RRs and lower SpO_2_s than those from lower elevations because of the lower partial pressure of oxygen.^6^, ^7^, ^8^, ^9^ Limited evidence is behind the development of the WHO thresholds, particularly when accounting for altitude.

Previous studies that were used to develop the WHO thresholds for RR were conducted across a range of altitudes from sea level to 1600 m, but did not include data from higher elevations.^10^, ^11^, ^12^, ^13^, ^14^ In the WHO algorithm, fast breathing is defined as at least 60 breaths per min in children aged 0–1 months, at least 50 breaths per min in children aged 2–11 months, and at least 40 breaths per min in children aged 12 months to 4 years. If a child has cough or difficulty breathing and is identified as having an elevated RR, guidelines recommend antibiotic treatment for potential pneumonia.^5^ The RR thresholds are intended for all children regardless of altitude, and use of the WHO guidelines at high altitudes could therefore lead to over-diagnosis of pneumonia in children with respiratory signs. More evidence is needed to delineate normal ranges of RR in children living at various elevations.

SpO_2_ measured at room air is a crucial criterion for classifying pneumonia severity.^15^ According to WHO guidelines, hypoxaemia (SpO_2_ of <90%, or <87% at altitudes greater than 2500 m) is an indicator of a child with severe pneumonia who requires supplemental oxygen.^16^ Hypoxaemia is associated with an increased risk of treatment failure and death in children in resource-limited countries, an effect that is exacerbated at high altitude.^17^, ^18^, ^19^, ^20^ Several studies have reported that the average SpO_2_ in healthy children is lower at higher altitudes than at sea level.^7^, ^8^, ^9^ However, WHO pneumonia management recommendations do not adequately account for altitude with the current thresholds.

We sought to determine the age-specific upper limit of normal for RR and lower limit of normal for SpO_2_ in healthy children in four countries at various altitudes, including moderate altitude (1000–2499 m above sea level) and high altitude (more than 2500 m above sea level). We expected mean RR to rise and mean SpO_2_ to decrease with higher altitudes. We also expected RR to fall with age, and for SpO_2_ to be lower during the early neonatal period than in older children (due to the transition from fetal to neonatal circulation), but to stabilise thereafter. We hypothesised that many healthy children would be misclassified as having abnormal RR or SpO_2_ according to WHO guidelines. We also report on the feasibility of using the Rad-97 automated respiratory rate counter in our different settings.

## Methods

### Study setting, design, and participants

We conducted this study in four sites in India, Guatemala, Rwanda, and Peru that are participating in the Household Air Pollution Intervention Network (HAPIN) trial, a large randomised controlled intervention trial designed to measure the effects of household air pollution on health outcomes in pregnant women, their offspring, and older adult women living in the same household. The study sites and their altitudes are listed in table 1.

**Table 1:** Participants by site, age, and sex

	**All regions**	**India (Nagapattinam and Villupuram, Tamil Nadu)**	**Guatemala (Jalapa District)**	**Rwanda (Kayonza District)**	**Peru (Puno Region)**
Average altitude (range), m	2175 (1–4348)	464 (1–919)	1362 (1036–2107)	1547 (1449–1644)	4088 (3827–4348)
Total	1570 (100%)	404 (25·7%)	389 (24·8%)	341 (21·7%)	436 (27·8%)
Female	796 (50·7%)	206 (51·0%)	195 (50·1%)	175 (51·3%)	220 (50·4%)
Male	774 (49·3%)	198 (49·0%)	194 (49·9%)	166 (48·7%)	216 (49·6%)
Age 0 to <2 months	609 (38·8%)	150 (24·6%)	147 (24·1%)	149 (24·5%)	163 (26·8%)
Age ≥2 to <12 months	519 (33·0%)	126 (24·3%)	119 (22·9%)	126 (24·3%)	148 (28·5%)
Age ≥12 to <24 months	442 (28·1%)	128 (29·0%)	123 (27·8%)	66 (14·9%)	125 (28·3%)

Data are n (%) unless otherwise specified. Age ranges were broken down by percentage of total participants per site.

The study was designed to determine mean RR and SpO_2_ values by age and location for healthy children in each of the four HAPIN sites. We also sought to test the feasibility of measuring RR using the Masimo Rad-97 pulse oximeter (Masimo Corporation, Irvine, CA, USA), which measures RR automatically via plethysmography. We aimed to enrol 400 healthy children at each site between Nov 24, 2017, and Oct 10, 2018 (150 children aged less than 2 months, 125 aged 2–11 months, and 125 aged 12–23 months). Children were recruited using a convenience sample from daycare centres, hospital-based nurseries for newborn babies, and outpatient health facilities where they presented for routine visits. Efforts were made to include both public and private facilities at each site.

Children were screened for eligibility via a structured survey administered to the parent and brief visual assessment of the child. Eligible children were younger than 24 months and resided within the study area. Exclusion criteria included parent-reported premature birth (<37 weeks gestation); parent-reported history of congenital heart defect; overnight admission to a health facility in the past 2 weeks; diagnosis of pneumonia; antibiotic use; respiratory signs (including cough, wheeze, phlegm, or shortness of breath); gastrointestinal signs (diarrhoea, loose stools, blood in stool, or oral rehydration treatment); self-reported history in the past 24 h of fever, difficulty breathing, fast breathing, runny nose, or nasal congestion; and runny nose, nasal congestion, fever, chest indrawing, or cyanosis as observed by study personnel. Written informed consent was obtained from a parent or guardian of eligible children.

Trained staff used the Rad-97 to obtain automated SpO_2_ and RR measurements, and then performed two manual measurements of RR. The study team developed a standard protocol with detailed instructions for collecting all measurements, both automated and manual, including photos and a video. The protocol was disseminated to each study site, and each site was responsible for local staff training. Rad-97 measurements were taken using either a foam wrap attachment on the base of the foot, or finger probe placed on the first finger or big toe. Selection of probe type and sensor placement followed manufacturer recommendations based on the child's age and size. The selected site was cleaned with an alcohol wipe. After sensor placement, the Rad-97 was allowed to stabilise (with uniform waveform and a valid measurement indicated on the device) for at least 1 min before taking readings, and only valid readings were recorded. Measurements were recorded while the child was calm, either awake or asleep.

Three consecutive SpO_2_ measurements were collected with the Rad-97 at 60, 90, and 120 s. Two automated RR measurements were collected at least 60 s apart. Field staff then immediately took two consecutive manual RR measurements, each over 60 s. Manual RR was measured by observing the child's naked chest for movement, counting one rise and fall of the chest as one breath. The same staff member did both automated and manual measurements and was therefore not masked to initial Rad-97 RR results. All sites were to collect both Rad-97 and manual RRs in a subset of 40 children for validation, and to collect automated RR only with the Rad-97 thereafter. However, several sites found it problematic to collect RR measurements with the Rad-97 because of the extra time needed to obtain a valid automated RR reading, which became infeasible for the study. Ultimately, we modified the study protocol to allow sites to obtain data with either the Rad-97 or manually.

### Average RR and SpO_2_ measurements

We reviewed all measurements for potential errors and excluded impossible values, such as SpO_2_ greater than 100%. One SpO_2_ value was less than 70%, and it was replaced with 70% as pulse oximetry cannot accurately measure SpO_2_ below 70%.^21^ Since SpO_2_ was obtained at 60 s, 90 s, and 120 s, we calculated the Pearson correlation between pairs of measurements and intracluster correlations (ICC) among all measurements.

We then calculated means of RRs measured by the Rad-97 (RR_Rad97_), manually (RR_manual_), or all RR measurements together (mean RR). Given that there is no gold standard for RR, we used mean RR to estimate smooth percentiles by age and site. We calculated mean SpO_2_ by averaging the three values obtained, and used this value to estimate smooth percentiles by age and site.

### Biostatistical methods

The primary analytical objective was to estimate percentile curves for mean RR and mean SpO_2_ by age and site and to compare with the WHO RR and SpO_2_ thresholds for tachypnoea and hypoxaemia. We based our statistical methods for the generation of smooth percentiles for RR and SpO_2_ by age and site based on the methods used to develop the WHO percentiles for child growth standards by age and site.^22^, ^23^ We used standard 2 × 2 table analyses to estimate precision, false positive and negative rates, and total misclassification rates for hypoxaemia and tachypnoea.

To estimate smooth percentile curves for RR and SpO_2_ by age and site, we built a generalised additive regression model for location, shape, and scale (GAMLSS)^24^ where either mean RR or SpO_2_ was used as the outcome, and age and site were used as covariates. We used the Box-Cox Power Exponential (BCPE) distribution, a highly flexible family of distributions that uses four parameters to describe location (μ, median), scale (σ, approximate coefficient of variation), skewness (ν, transformation to symmetry), and kurtosis (τ, power exponential parameter).^22^, ^23^, ^24^ We found that the best distribution to fit respiratory rate and oxyhaemoglobin saturation was the BCPE distribution (appendix pp 1–2). We modelled each parameter of the BCPE distribution with a penalised B-spline for age, indicator variables for site, and a penalised varying coefficient for the interaction between age and site for both RR and SpO_2_ (appendix p 2). We used a generalised Akaike Information Criterion (AIC) as a measure of goodness-of-fit to determine how many of the parameters of the BCPE distribution were required. The model that used all four parameters of the BCPE distribution had the lowest AIC (appendix p 1). We calculated percentiles for RR (50th, 90th, and 95th) and SpO_2_ (5th, 10th, and 50th) by age and site using the final GAMLSS regression model. We assessed goodness-of-fit using residual plots and found that the models appropriately fit the data (appendix p 9).

We calculated false positive and false negative rates and total misclassification rates for tachypnoea if we used the WHO recommended thresholds instead of the 95th percentile cutoffs by age and site (appendix p 10). We did similar calculations for hypoxaemia if we used the WHO thresholds (<90% at altitudes ≤2500 m and <87% above 2500 m) instead of the 5th percentile cutoffs by age and site. We calculated both site-specific and overall misclassification rates. Given uncertainties in the estimated percentile functions from the GAMLSS fit, we calculated 95% bootstrap CIs for the misclassification rates using 50 bootstrap samples.

We did two types of sensitivity analyses. First, we calculated false positive and false negative rates and total misclassification rates for tachypnoea if the 90th percentile was used for RR, and for hypoxaemia if the 10th percentile was used for SpO_2_. Second, we also calculated false positive and false negative rates and total misclassification rates for tachypnoea (or hypoxaemia) if either RR_Rad97_ or RR_manual_ was used alone.

In secondary analyses, we estimated agreement between the two RR measurements obtained using the Masimo Rad-97 and those obtained manually, and also estimated agreement between the mean values obtained from each method (mean RR_Rad97_ *vs* mean RR_manual_). We used the Bland-Altman method to estimate agreement.^25^ The differences between mean RR_Rad97_ and mean RR_manual_ were right-skewed.

However, as previously noted by Bland and Altman,^25^ a non-normal distribution of differences might not be as serious when using the Bland-Altman approach as in other statistical contexts. Following further recommendations by Bland and Altman, we also calculated non-parametric limits of agreement by using the 5th and 95th percentiles of the differences in RRs. All analyses were done in R and the GAMLSS package.

### Ethics review

The study protocol was reviewed and approved by the ethics committees at Johns Hopkins University (00007464), Emory University (00089799), Sri Ramachandra Institute of Higher Education and Research (IEC-N1/16/JUL/54/49) and the Indian Council of Medical Research–Health Ministry Screening Committee (5/8/4-30/(Env)/Indo-US/2016-NCD-I), Universidad del Valle de Guatemala (146-08-2016/11-2016) and Guatemalan Ministry of Health National Ethics Committee (11-2016), A.B. PRISMA (CE3571.16), the London School of Hygiene and Tropical Medicine (11664-5) and the Rwandan National Ethics Committee (No.357/RNEC/2018), and Washington University in St Louis (201611159). Our study was formative work for the HAPIN trial, which is registered with ClinicalTrials.gov (Identifier NCT02944682).

### Role of the funding source

The funders of the study made recommendations for the study design but final decisions were made independently by study investigators. The funders had no role in the data collection, analysis, or interpretation, in the writing of the report, or the decision to submit the paper for publication. The corresponding author had full access to the data in the study and had final responsibility for the decision to submit for publication.

## Results

Between Nov 24, 2017, and Oct 10, 2018, 2027 children were screened for eligibility at centres in India, Guatemala, Peru, and Rwanda. 1692 met eligibility criteria. 30 participants were excluded due to missing values (seven without sex recorded, four without SpO_2_, and 19 without RR), and 92 participants were subsequently excluded due to measurement or data entry errors, leaving 1570 children included in the final analysis (appendix p 11).

We analysed data for 404 participants from India, 389 from Guatemala, 341 from Rwanda, and 436 from Peru. Average ages were similar between India (7·7 months, SD 7·4), Guatemala (7·8 months, 7·5) and Peru (7·4 months, 7·3) but lower in Rwanda (5·5 months, 5·9). 51% of participants were girls, and there was no appreciable difference in the proportion of girls across sites (p=0·97; table 1).

**Figure 1:**
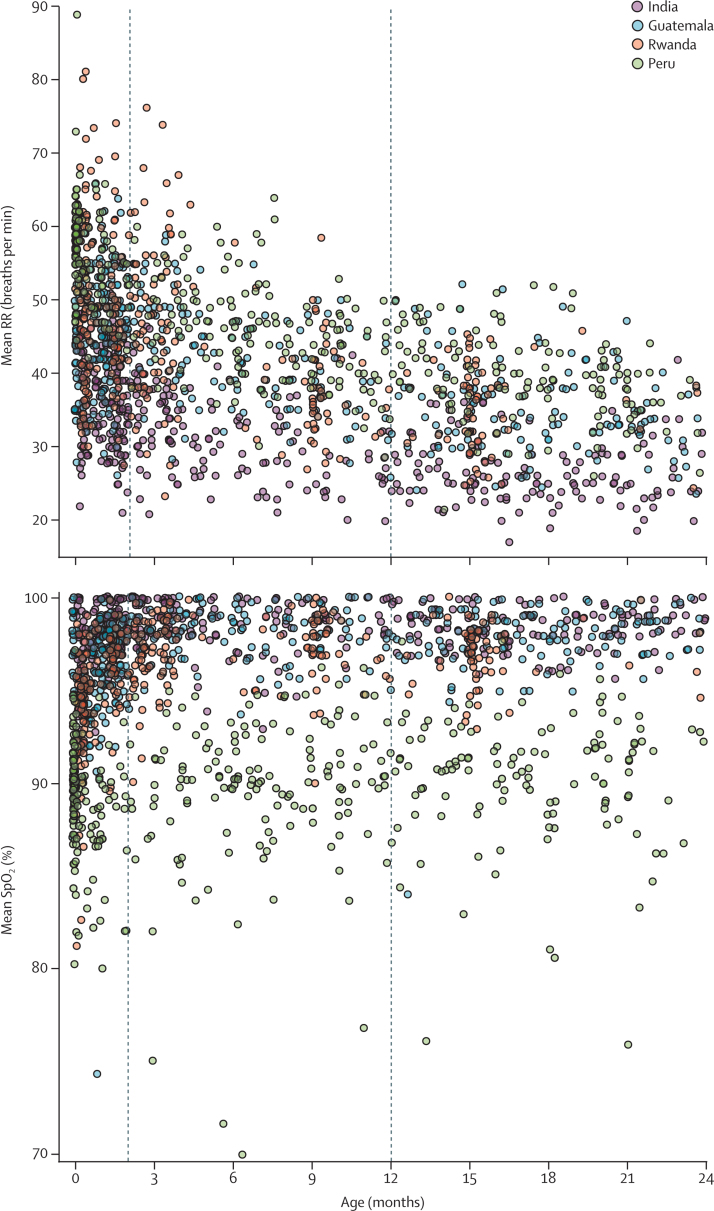
Mean RR and SpO_2_ by age in months, stratified by site RR=respiratory rate. SpO_2_=oxyhaemoglobin saturation. Each observation is represented by a coloured circle based on site.

Mean RR and SpO_2_ by age and site are shown in figure 1. Mean RR was highest in Peru (altitude 3827–4348 m), followed by Rwanda (1449–1644 m), Guatemala (1036–2107 m), and India (1–919 m), tracking with elevation (from highest to lowest). Mean RR was 31·9 breaths per min in India (SD 7·1), 41·5 breaths per min in Guatemala (8·4), 44·0 breaths per min in Rwanda (10·8), and 48·0 breaths per min in Peru (9·4). Mean SpO_2_ was 98·3% in India (SD 1·5), 97·3% in Guatemala (2·4), 96·2% in Rwanda (2·6), and 89·7% in Peru (3·5). Compared to India, mean RR was 9·6 breaths per min higher in Guatemala, 12·1 breaths per min higher in Rwanda, and 16·1 breaths per min higher in Peru (likelihood ratio test p<0·0001). Age was an important determinant of mean RR (p<0·0001) and RR was lower with older age at all percentiles and for all sites.

**Figure 2:**
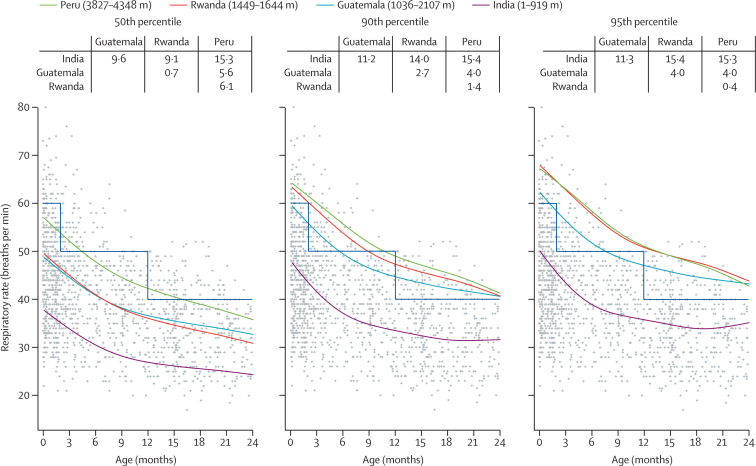
50th, 90th, and 95th percentiles by age and site for mean RR RR=respiratory rate. Estimated percentiles obtained from a generalised additive regression model for location, shape, and scale fitted to RR data. The observed data points for all settings are plotted in grey. Smooth lines represent site-specific estimated percentiles by age. The blue step line indicates the thresholds recommended by WHO for determining abnormal RR. The table at the top of each panel summarises the mean difference in RR between pairs of countries.

50th, 90th, and 95th smooth percentiles for mean RR by age and site, and average differences between estimated percentiles are shown in figure 2. The percentile curves show greater mean RR with higher altitude, with India being the lowest, Guatemala and Rwanda being intermediate, and Peru the highest. These differences were fairly consistent across percentiles.

Figure 2 shows the WHO thresholds for RR to allow visualisation of the extent of misclassification if these cutoffs were applied to children with respiratory signs in our study population. We provide site-specific plots in the appendix (p 12). The 95th percentile for RR for Peru and Rwanda fell above the WHO cutoff, that for Guatemala overlapped with it, but that for India fell considerably below it. The 95th percentile curves for Peru and Rwanda were quite similar despite a large difference in altitude. When we compared the smooth 95th percentile for RR by age and site against the WHO-defined RR thresholds, the false positive rates for tachypnoea were 0% in India, 7·3% in Guatemala, 16·8% in Rwanda, and 28·9% in Peru (table 2). We found similar results when using the subset of children aged 2–11 months (appendix p 5).

**Table 2:** Misclassification rates for tachypnoea when using mean RR

	Children with RR ≥ percentile and WHO upper threshold*(true positives; a)	Children with RR ≥ WHO upper threshold*but < percentile (false positives; b)	Children with RR ≥ percentile but < WHO upper threshold*(false negatives; c)	Children with RR < percentile and WHO upper threshold*(true negatives; d)	**False positives for tachypnoea, % (95% bootstrap CI)**	**False negative for tachypnoea, % (95% bootstrap CI)**	**Total misclassification, % (95% bootstrap CI)**
**95th percentile**							
India	1	0	21	382	0·0 (0–0)	95·5% (87·4–100)	5·2% (3·2–6·9)
Guatemala	20	27	0	342	7·3% (4·1–10·4)	0·0 (0–9·7)	6·9% (4·0–9·9)
Rwanda	19	54	0	268	16·8% (12·9–21·1)	0·0 (0–1·7)	15·8% (12·1–20·2)
Peru	14	122	0	300	28·9% (23·7–33·0)	0·0 (0–1·9)	28·0% (22·6–31·9)
**90th percentile**							
India	1	0	42	361	0·0 (0–0)	97·7% (92·9–100)	10·4% (7·3–12·4)
Guatemala	33	14	7	335	4·0% (1·8–6·5)	17·5% (0·8–36·8)	5·4% (3·6–7·8)
Rwanda	32	41	0	268	13·3% (8·8–17·7)	0·0 (0–8·4)	12·0% (8·1–16·3)
Peru	31	105	0	300	25·9% (20·4–30·1)	0·0 (0–7·5)	24·1% (18·6–28·0)

RR=respiratory rate. Data are n, unless otherwise specified.

* WHO upper threshold ≥60 breaths per min for children 0–1 months, ≥50 breaths per min for children 2–11 months, ≥40 breaths per min for children 12–23 months. We define the false positive rate for tachypnoea as b/(b + d), the false negative rate for tachypnoea as c/(a + c), and the total misclassification rate for tachypnoea as (b + c)/(a + b + c + d).

**Figure 3:**
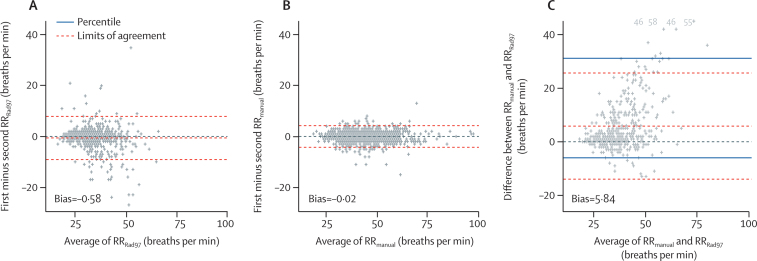
Bland-Altman plot showing agreement between RRs RR=respiratory rate. (A) Agreement between first and second Rad-97 RRs. (B) Agreement between first and second manual RRs. (C) Agreement between mean manual RR and mean Rad-97 RR. We plotted both the limits of agreement using the standard Bland-Altman method and a non-parametric percentile method using the 5th and 95th percentiles. The four values above 40 breaths per min in (C) are plotted in grey at the bottom of the panel and aligned horizontally.

In the secondary analysis, the Bland-Altman method showed the extent of agreement within RR_Rad97_ measurements, within RR_manual_ measurements, and between RR_Rad97_ and RR_manual_ measurements (figure 3). The mean of the differences within the two RR_Rad97_ measurements was close to zero, as was the mean of the differences within the two RR_manual_ measurements. There was greater agreement within RR_manual_ measurements, with limits of agreement close to 5 breaths per min, whereas the limits of agreement for RR_Rad97_ were closer to 10 breaths per min. The mean difference between RR_manual_ and RR_Rad97_ was higher than RR_manual_ at 5·8 breaths per min with higher mean manual rates. Differences between RR_manual_ and RR_Rad97_ were greater in children with higher RRs. Given the appreciable difference in agreement between approaches to measure RR, we recalculated false positive and negative rates for tachypnoea and total misclassification rate if RR_Rad97_ or RR_manual_ were used alone, but did not find major differences in these rates when compared to those obtained when using mean RR (appendix pp 6, 7).

**Figure 4:**
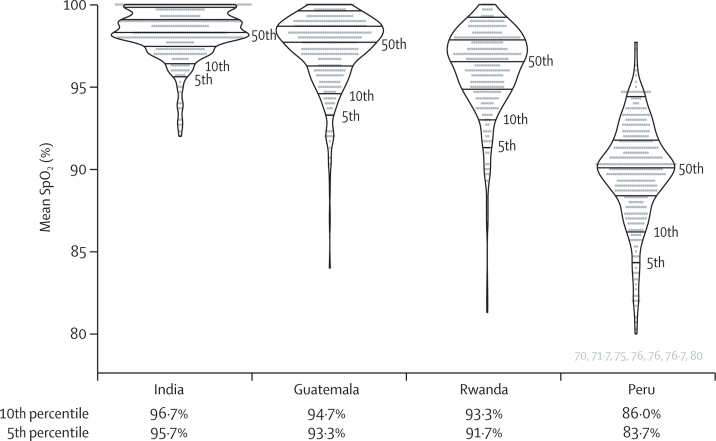
Violin plot showing the 5th, 10th, and 50th percentiles for SpO_2_ in children of all ages by site Horizontal lines inside the modified boxplots represent the 5th, 10th, 25th, 50th, 75th, and 90th percentiles. The seven SpO_2_ values 80% or lower in Peru are plotted in grey below its violin plot. SpO_2_=oxyhaemoglobin saturation.

SpO_2_ was higher at low altitudes and lower at high altitudes (figure 1). There were large variations between the 5th, 10th, and 50th percentiles (figure 4). Mean SpO_2_ was 98·3% in India (SD 1·5), 97·3% in Guatemala (2·4), 96·2% in Rwanda (2·6), and 89·7% in Peru (3·5). Compared to India, mean SpO_2_ was 1% lower in Guatemala, 2·1% lower in Rwanda, and 8·6% lower in Peru (p<0·0001. Age was also an important determinant of mean SpO_2_ (p<0·0001), and mean SpO_2_ was higher with older age especially for the 5th percentiles.

**Figure 5:**
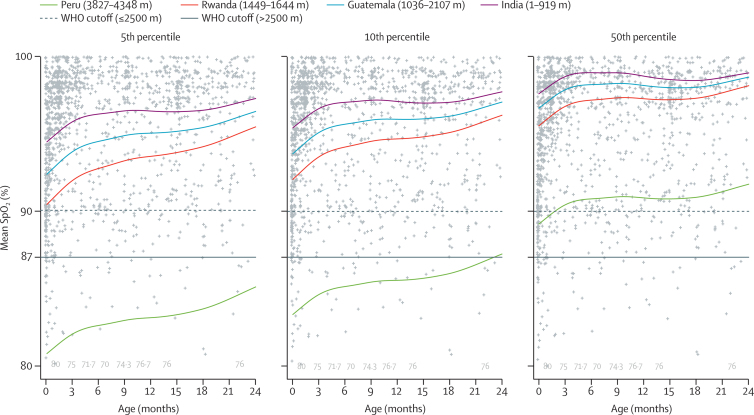
50th, 90th, and 95th percentiles by age and site for mean SpO_2_ SpO_2_=oxyhaemoglobin saturation. Estimated percentiles were obtained from a generalised additive regression model for location, shape, and scale fitted to SpO_2_ data. We plotted the observed data points in grey. Smooth lines represent site-specific percentiles by age. The seven SpO_2_ values 80% or lower in Peru are plotted in grey by age at the bottom of the panel.

Estimated 5th, 10th, and 50th smooth percentiles for SpO_2_ by age and site are shown in figure 5. We provide site-specific plots in the appendix (p 13). The percentile lines for India, Guatemala, and Rwanda clustered closely together, whereas those for Peru were lower. The 50th percentile lines for the three lower altitude countries were in the mid to high 90s, whereas that for Peru was just above 90%. The 5th percentile line for Peru was at 81–86%. Saturations rose after birth, plateaued temporarily, and then increased again around 18 months of age. When using the WHO-defined SpO_2_ cutoffs of less than 90% for India, Guatemala, and Rwanda, and less than 87% for Peru, we found that false positive rates for hypoxaemia among children whose SpO_2_ was below the 5th percentile was 0% in India, 0% in Guatemala, 0% in Rwanda, and 11·6% in Peru (table 3). We found similar results when using the subset of children aged 2–11 months (appendix p 8).

**Table 3:** Misclassification rates for hypoxaemia

	Children with SpO_2_ < percentile and WHO lower threshold*(true positives; a)	Children with SpO_2_ < WHO lower threshold*but ≥ percentile (false positives; b)	Children with SpO_2_ < percentile but ≥ WHO lower threshold*(false negatives; c)	Children with SpO_2_ ≥ percentile and WHO upper threshold*(true negatives; d)	**False positives for hypoxaemia, % (95% bootstrap CI)**	**False negatives for hypoxaemia, % (95% bootstrap CI)**	**Total misclassification, % (95% bootstrap CI)**
**5th percentile**							
India	0	0	24	380	0·0 (0–0)	100·0% (100–100)	5·9% (4·1–7·3)
Guatemala	4	0	22	363	0·0 (0–0)	84·6% (65·1–100)	5·7% (3·0–7·5)
Rwanda	8	0	11	322	0·0 (0–0.4)	57·9% (36·6–85·5)	3·2% (1·3–5·3)
Peru	13	49	0	374	11·6% (7·0–14·7)	0·0 (0–0)	11·2% (6·7–14·2)
**10th percentile**							
India	0	0	49	355	0·0 (0–0)	100·0% (100–100)	11·4% (8·2–12·3)
Guatemala	4	0	44	341	0·0 (0–0)	91·7% (83·5–100)	11·3% (9·0–13·2)
Rwanda	8	0	28	305	0·0 (0–0)	77·8% (67·7–93)	8·2% (5·7–11·5)
Peru	34	28	0	374	7·0% (2·4–10)	0·0 (0–8·5)	6·4% (2·3–9·4)

Data are n, unless otherwise specified. SpO_2_ =oxyhaemoglobin saturation.

* WHO lower threshold <90% at altitudes ≤2500 m and <87% at altitudes above 2500 m. Here, we define a false positive rate for hypoxaemia as b/(b + d), false negative rate for hypoxaemia as c/(a + c), and total misclassification rate for hypoxaemia as (b + c)/(a + b + c + d).

**Figure 6:**
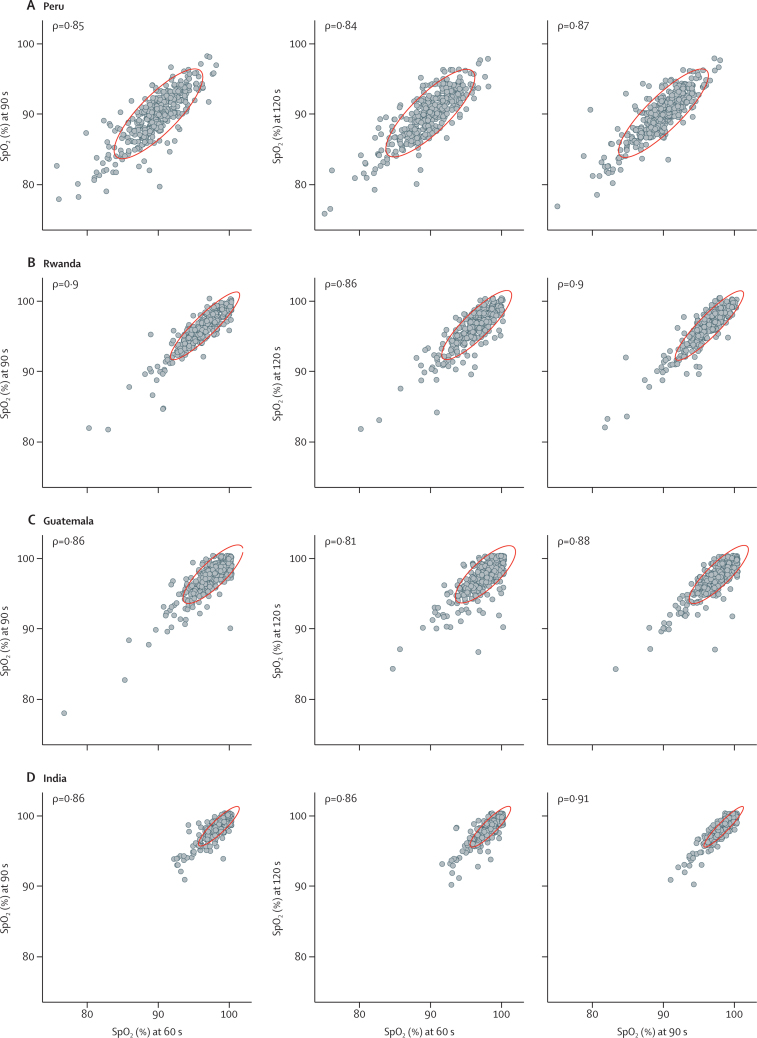
Pearson correlations between consecutive SpO_2_ measurements SpO_2_=oxyhaemoglobin saturation. Scatterplots are provided of SpO_2_ at 60 versus 90 s, 60 versus 120 s, and 90 versus 120 s for each site, with associated Pearson correlation coefficients (ρ). The red ellipses represent 95% CI for the data in each panel.

The Pearson correlation between pairs of consecutive measurements of oxyhaemoglobin saturation with the Rad-97 showed high correlation between measurements for all sites (figure 6). Estimated ICCs were similar for India (ICC 0·88, 95% CI 0·86–0·89), Guatemala (0·84, 0·82–0·87), Rwanda (0·89, 0·87–0·90), and Peru (0·86, 0·83–0·87).

## Discussion

We examined the distribution of RR and SpO_2_ of healthy children younger than 2 years in four low-resource settings at different altitudes. We found that the 95th percentile curves for RR and 5th percentile curves for SpO_2_ by age followed a general pattern of increasing RR and decreasing SpO_2_ with increasing altitude.

The WHO-defined thresholds resulted in misclassification of a large proportion of the healthy children in our sample as having tachypnoea or hypoxaemia, particularly at high altitudes. Based on this finding, children living at high altitude who present for care with upper respiratory signs, and without lower respiratory tract infection, could also be misclassified as tachypnoeic or hypoxaemic using the same thresholds. According to WHO Integrated Management of Childhood Illness (IMCI) guidelines, the presence of tachypnoea in children with respiratory signs is an indicator of pneumonia, so this false classification could lead to unnecessary and potentially harmful treatment.

These results reinforce previous findings about respiratory physiology at altitude.^6^ However, it is worth focusing on the implications considering the commonly used WHO IMCI algorithm, which uses fast breathing for age as one of the key signs for identification of pneumonia. Our comparisons of the 95th percentiles for RR with the WHO cutoffs show increasing false positive rates and misclassification for tachypnoea with altitude, reaching an alarming rate in Peru. This misclassification could lead to over-diagnosis of pneumonia and thus overuse of antibiotics and other resources in children with respiratory signs. Conversely, the 95th percentile line for RR in India fell well below the WHO cutoff, indicating that the WHO cutoff might have inadequate sensitivity to detect tachypnoea there. For SpO_2_, many children at higher altitudes would be misclassified as hypoxaemic using the WHO guidelines, even those specified for use at altitudes above 2500 m. This could result in erroneous designation of children with minor illnesses as having more severe disease, and lead to unnecessary hospital admission or treatment. Our findings show a variation in RR and SpO_2_ at sites of various altitudes that is not unexpected but might defy neat categorisation into a one-size-fits-all approach such as the WHO guidelines.

We also had an opportunity to assess the feasibility of using an automated device to measure RR. Both manual counting and the Rad-97 showed reasonable internal consistency with repeated measurements. However, the Rad-97 reported a lower RR than did manual assessments (an error that became more pronounced at higher RRs). This was unexpected, given that the manual measurements were done last, and led us to conclude that the Rad-97 showed poor performance at higher respiratory rates. Moreover, field staff found the Rad-97 infeasible for use in young children as it required several minutes to reach a valid, uniform plethysmography waveform, which was attributed to participant movement interfering with the signal. Manual RR was the preferred method of assessment by our study team.

Our study has several strengths. First, we included four sites at across a range of altitudes using consistent equipment and protocols. Most previous studies of RR or SpO_2_ in healthy children at altitude have examined only one or two sites and vary in methods between studies, making drawing conclusions across studies difficult and prone to error. By contrast, our method allows for more reliable conclusions about the differences between altitudes. Second, we have a large sample size over a narrow age range, with good representation in the first few months of life when RR falls and SpO_2_ rises most rapidly. This rise in SpO_2_ is a consequence of switching from fetal haemoglobin to adult haemoglobin. Our data, however, revealed a slower rise in SpO_2_ than expected during the early neonatal period. Substantiation of this finding by future studies would mean WHO thresholds need to be appropriately adjusted for use in neonates.

There are some potential limitations. First, our study determined centiles for RR and SpO_2_ in apparently healthy children without respiratory signs. However, the WHO algorithm for diagnosis of pneumonia is intended for use in children with respiratory signs, who we excluded from our study. To verify the conclusion that the WHO criteria will misclassify children as having pneumonia, the thresholds determined from percentiles need to be validated in children with cough and difficulty breathing. Second, we use parent-report to document conditions that could lead to chronic hypoxaemia, such as congenital heart disease. Third, we did all RR_Rad97_ measurements consistently before RR_manual_. Future designs could use a crossover approach, or preferably one in which the measurements are obtained in parallel, or there is a video reference panel.

Our study used a convenience sample of children presenting to health-care facilities and daycare centres, and therefore might not be generalisable to a larger population. Manual RR counting was always done after automated RR measurement and by the same staff member. It is possible that any interaction with the child to place the Masimo sensor could have caused anxiety and systematic elevation in the RR for all children. However, each site followed the same protocol, and differences between sites should be maintained. Staff were not masked to the automated RR result, which might have influenced their manual RR result. We did not document whether the child was sleeping or awake during measurements, so we cannot be sure whether this general state influenced differences between sites. All four sites used the same highly detailed protocol, but they were each in charge of training their own staff, which might have led to inconsistencies during implementation. Indeed, one of the four countries had outliers at first that were explained by errors in protocol interpretation and were excluded from our analysis.

In addition, because this was formative work undertaken to inform a larger trial, we were unable to account for some factors that could have influenced differences in RR or SpO_2_ between our study sites. For example, we did not account for genetic variations in adaptation to living at altitude. Our samples from each country could have included children from various ethnicities, who might respond differently to altitude in terms of both RR and SpO_2_. We also did not measure or control for anaemia, which is common and varies in prevalence between our sites.^26^, ^27^, ^28^, ^29^ Low haemoglobin concentration in anaemia results in increased RR,^30^ so we cannot be sure that some of the differences in RR between sites are not due to differences in prevalence of anaemia; likewise with nutritional status or body size. Finally, the degree of exposure to household air pollution could also have influenced our outcomes of interest but was not measured in this study.

The WHO guidelines for SpO_2_ and RR have been used to develop definitions of pneumonia widely used in both clinical and research settings. Appropriate pneumonia definitions are crucial for proper triage of ill children, appropriate treatment, and suitable apportionment of scarce resources such as supplemental oxygen. In pneumonia intervention trials, a highly specific pneumonia case definition is essential for detection of intervention effects.^31^ Therefore, it is imperative that pneumonia definitions using SpO_2_ and RR use the most accurate thresholds possible. Our study shows that a one-size-fits-all approach, as is recommended by WHO IMCI definitions of fast breathing for age and hypoxaemia, could result in misclassification of a substantial proportion of children who present with respiratory signs at moderate to high altitudes. Using predictive modelling, we were able to generate the upper limit of normal RR and lower limit of normal SpO_2_ by age in each of our four study sites. These models could be adopted to guide pneumonia diagnosis and management and reduce rates of misclassification, and similar predictive curves could be generated and implemented in other areas with high pneumonia mortality to increase effectiveness of both clinical care and research.
