# Supplementary material for: Effects of high altitude on respiratory rate and oxygen saturation reference values in healthy infants and children younger than 2 years in four countries: a cross-sectional study
Source: Lancet Glob Health. 2020 Feb 19;8(3):e362–73. doi: 10.1016/S2214-109X(19)30543-1 (PMC7034060; doi:10.1016/S2214-109X(19)30543-1)

THE LANCET
Global Health

Supplementary appendix

This appendix formed part of the original submission and has been peer reviewed. We post it as supplied by the authors.

Supplement to: Crocker ME, Hossen S, Goodman D, et al. Effects of high altitude on respiratory rate and oxygen saturation reference values in healthy infants and children younger than 2 years in four countries: a cross-sectional study. *Lancet Glob Health* 2020; **8:** e362–73.

Online Supplement for "Effects of high altitude on respiratory rate and oxygen saturation reference values in healthy infants and children under two years of age in four countries"

Model selection

Generalized additive models for location, shape, and scale allow the use of several distributions for fitting continuous data. Since many of these distributions do not belong to the same family, we used a generalized Akaike Information Criterion (AIC) to select the distribution that best fit our data. We found that the best distribution to fit respiratory rate and oxyhaemoglobin saturation was the Box-Cox Power Exponential (BCPE) distribution, a highly flexible family of distributions that uses four parameters to describe location (μ, median), scale (σ, approximate coefficient of variation), skewness (ν, transformation to symmetry), and kurtosis (τ, power exponential parameter).

e-Table 1. Results from fitting generalized additive models for location, shape, and scale to mean respiratory rate measurements in 1570 children.

**Distribution**	**Generalized Akaike Information Criterion**	
	**Modelling the location parameter (μ) using only an intercept**	**Modelling the location parameter (μ) using a penalized B-spline for age**
Box-Cox power exponential	11862	11309
Gamma	11879	11336
Generalized Gamma	11881	11320
Box-Cox Cole and Green	11882	11328
Box-Cox t	11884	11330
Power exponential	11930	11399
Normal	11937	11400
Truncated t	11939	11402

e-Table 2. Results from fitting generalized additive models for location, shape, and scale to mean oxyhaemoglobin saturation measurements in 1570 children.

**Distribution**	**Generalized Akaike Information Criterion**	
	**Modelling the location parameter (μ) using only an intercept**	**Modelling the location parameter (μ) using a penalized B spline for age**
Box-Cox power exponential	6529	6472
Gamma	9142	9050
Generalized Gamma	Did not converge	Did not converge
Box-Cox Cole and Green	Did not converge	Did not converge
Box-Cox t	Did not converge	Did not converge

Power exponential	9054	Did not converge
Normal	9073	8978
Truncated t	9005	8849

Once we selected the BCPE as the best distribution to fit our data, we proceeded to identify the most parsimonious model by fitting combinations of penalized B-splines for age (cs[age]), indicator variables for site (factor[site]), and penalized varying coefficients for the interaction between age and site (PVC[age, site]) in each of the four parameters of the BCPE distribution. To achieve this, we constructed sequential models whereby we included penalized B-splines for age, indicator variables for site, and penalized varying coefficients for the interaction between age and site into μ, σ, ν, and τ. We also used the generalized AIC to identify the most parsimonious models. In e-Table 3, we show that the best model to fit mean respiratory rate measurements with the lowest generalized AIC was one that had a smooth term of age, a factor for site and a varying coefficient for the interaction between age and site in all four parameters of the BCPE distribution. In e-Table 4, we show that the best model to fit mean oxyhaemoglobin saturations measurements one with a smooth term of age, a factor for site and a varying coefficient for the interaction between age and site in μ and σ. Models for oxyhaemoglobin saturation where we parametrized ν and τ using the covariates did not converge.

e-Table 3. Model building for respiratory rate using the four parameters of the Box-Cox Power Exponential distribution.

**Parameters**	**Generalized Akaike Information Criterion**
**Modelling μ, σ, ν and τ** with cs(age) + factor(site) + PVC(age, site).	10292
**Modelling μ, σ and ν** with cs(age) + factor(site) + PVC(age, site) and **τ** with an intercept only.	10305
**Modelling μ and σ** with cs(age) + factor(site)+ PVC(age, site) and **ν an d τ** with an intercept only.	10313
**Modelling μ** with cs(age) + factor(site) + PVC(age, site) and **σ, ν and τ** with an intercept only.	10414

e-Table 4. Model building for oxyhaemoglobin saturation using the four parameters of the Box-Cox Power Exponential distribution.

**Parameters**	**Generalized Akaike Information Criterion**
**Modelling μ and σ** with cs(age) + factor(site) + PVC(age, site) and **ν and τ** with an intercept only.	6550
**Modelling μ** with cs(age) + factor(site) + PVC(age, site) and **σ, ν an d τ** with an intercept only.	6806

Assessment of goodness-of-fit

We constructed worm plots of the residuals for fitted models of respiratory rate and oxyhaemoglobin saturation in e-Figure 1. The worm plot for respiratory rate shows that all points are close to the horizontal indicating that the BCPE distribution and fitted model provide an appropriate fit to the data. The worm plot for oxyhaemoglobin saturation shows that all points but three are close to the horizontal, also indicating that the BCPE distribution and fitted model provide a reasonable fit to the data.

Assessment of misclassification

We assessed for misclassification by calculating 2 x 2 tables (e-Figure 2). We calculated false positive and false negative rates and total misclassification rates for tachypnoea if we used the WHO recommended thresholds instead of the age- and site-specific 95th (or 90th) percentile cutoffs. We performed similar calculations for hypoxaemia if we used the WHO thresholds (<90% at altitudes ≤2,500 m and <87% above 2,500 m) instead of the age- and site-specific 5th (or 10th) percentile cutoffs. We calculated both site-specific and overall misclassification rates. Given uncertainties in the estimated percentile functions from the GAMLSS fit, we calculated 95% bootstrap CIs for the misclassification rates using 50 bootstrap samples (e.g., for false positive [FP] rate, we calculated the 95% CI as $FP \pm 1.96 \times \hat{\sigma}_{50\ boostrapped\ FPs}$).

Analytical sample

Of 2,027 children screened for eligibility, 1,692 met eligibility criteria and were included in our analysis. During data analysis, 30 participants were dropped due to missing values (seven without sex recorded, four without SpO_2, and 19 without RR). Additionally, 92 participants were dropped due to measurement error. This left 1,570 children in the final analysis (e-Figure 3).

Upper limit of normal age-specific percentile curves for each site

In e-Figure 4, we show site-specific data for respiratory rate by age and contrast the World Health Organization cutoff for tachypnoea with the estimated age-specific 95th smooth percentile by site. Our analyses revealed that respiratory rate was higher at birth and was lower with older age in children at all sites. As hypothesized, respiratory rate was positively correlated with altitude, so that children living at higher altitudes had higher mean respiratory rates.

In e-Figure 5, we show site-specific data for respiratory rate by age and contrast the World Health Organization cutoff for hypoxaemia with the estimated age-specific 5th smooth percentile by site. SpO_2 also followed the expected pattern in that it was inversely correlated with altitude. Furthermore, the lowest SpO_2s at each site were measured in newborn infants, reflecting the transition from the foetal to neonatal circulation.

Sensitivity analyses for tachypnoea in the subset of children aged 2-11 months

We conducted a sensitivity analysis in which we limited our sample of data to children 2-11 months of age and found similar results when assessing misclassification for tachypnoea (e-Table 5) when compared to children in the entire cohort.

Sensitivity analyses to calculate misclassification rates for tachypnoea if only Masimo Rad97 or manual respiratory rate measurements were used

We calculated false positive and negative rates and total misclassification rates for tachypnoea (or hypoxaemia) if either RR_{Rad97} or RR_{manual} were used alone to measure RR. On average, RR_{manual} was 0·5, 0·7, 2·6, 0·2 breaths/min higher than mean RR whereas mean RR_{Rad97} was 0·1, 3·3, 3·6, 2·4 breaths/min lower than mean RR in India, Guatemala, Rwanda, and Peru, respectively. We used these values to shift the percentile curves up or down and recalculated false positive and negative rates for tachypnoea and

total misclassification rate. The results for RR_{manual} are shown in e-Table 6, and those for RR_{Rad97} are shown in e-Table 7. There was no appreciable difference in false positive or negative rates and total misclassification when RR_{manual} was used when compared to values obtained when mean RR was used. When RR_{Rad97} was used, total misclassification rates were similar whereas false positive rates were lower in magnitude but in the same direction of higher rates tracking with elevation when compared to values obtained when mean RR was used.

Sensitivity analyses for hypoxemia in the subset of children aged 2-11 months

We conducted a sensitivity analysis in which we limited our sample of data to children 2-11 months of age and found similar results when assessing misclassification for hypoxaemia (e-Table 8) when compared to children in the entire cohort.

e-Table 5. Misclassification rates for tachypnoea when using mean respiratory rate in children aged 2-11 months. In this table, **a** is the number of children with a RR ≥ 95th percentile and WHO upper thresholds (≥50 breaths/min for children 2-11 months); **b** is the number of children with a RR ≥ WHO upper threshold but below the 95th percentile; **c** is the number of children with a RR ≥ 95th percentile but below the WHO upper threshold; and **d** is the number of children with a RR below 95th percentile and WHO upper threshold. In this table, a = true positives, b = false positives, c = false negatives, and d = true negatives. Here, we define a false positive rate for tachypnoea (using the 95th percentile as the reference standard) as $\frac{b}{b+d}$, a false negative rate for tachypnoea as $\frac{c}{a+c}$, and the total misclassification rate for tachypnoea as $\frac{b+c}{a+b+c+d}$. We repeated the same calculations if we used 90th percentile of RR instead.

		**a =** Number of children with RR ≥ percentile and WHO upper thresholds	**b =** Number of children with RR ≥ WHO upper threshold but < percentile	**c =** Number of children with RR ≥ percentile but < WHO upper threshold	**d =** Number of children with RR below percentile and WHO upper thresholds	% false positive for tachypnoea (95% bootstrap CI)	% false negative for tachypnoea (95% bootstrap CI)	% total misclassification (95% bootstrap CI)
**95th percentile**								
	India	0	0	5	121	0 (0 – 0)	100 (100 - 100)	4 (1 - 7·5)
	Guatemala	7	9	0	103	8 (3·2 - 13·2)	0 (0 - 26·5)	7·6 (3·2 - 13·0)
	Rwanda	10	24	0	92	20·7 (13·7 - 29·9)	0 (0 - 0)	19 (12·6 - 27·6)
	Peru	6	34	0	108	23·9 (16·8 - 31·9)	0 (0 - 0)	23 (15·9 - 31·0)
**90th percentile**								
	India	0	0	8	118	0 (0 - 0)	100 (100 - 100)	6·3 (3·2 - 10·2)
	Guatemala	9	7	4	99	6·6 (1·2 - 9·9)	30·9 (0 - 63·2)	9·2 (2·7 - 14·0)
	Rwanda	16	18	0	92	16·4 (9·1 - 25·3)	0 (0 - 14·1)	14·3 (8·6 - 22·1)
	Peru	10	30	0	108	21·7 (14 - 29·8)	0 (0 - 20·5)	20·3 (13·1 - 28·3)

e-Table 6. Misclassification rates for tachypnoea when using manual respiratory rate only. In this table, **a** is the number of children with a RR ≥ 95th percentile and WHO upper thresholds (≥60 breaths/min for children 0-1 months, ≥50 breaths/min for children 2-11 months, ≥40 breaths/min for children 12-23 months); **b** is the number of children with a RR ≥ WHO upper threshold but below the 95th percentile; **c** is the number of children with a RR ≥ 95th percentile but below the WHO upper threshold; and **d** is the number of children with a RR below 95th percentile and WHO upper threshold. In this table, a = true positives, b = false positives, c = false negatives, and d = true negatives. Here, we define a false positive rate for tachypnoea (using the 95th percentile as the reference standard) as $\frac{b}{b+d}$, a false negative rate for tachypnoea as $\frac{c}{a+c}$, and the total misclassification rate for tachypnoea as $\frac{b+c}{a+b+c+d}$. We repeated the same calculations if we used 90th percentile of RR instead.

	**a =** Number of children with RR ≥ percentile and WHO upper threshold	**b =** Number of children with RR ≥ WHO upper threshold but < percentile	**c =** Number of children with RR ≥ percentile but < WHO upper threshold	**d =** Number of children with RR < percentile and WHO upper threshold	% false positive for tachypnoea	% false negative for tachypnoea	% total misclassification
**95th percentile**							
**India**	1	0	18	385	0 (0 - 0·1)	94·7 (8·08 - 100)	4·5 (3·5 - 6·9)
**Guatemala**	17	30	0	342	8·1 (4·0 - 10·4)	0 (0 - 5·3)	7·7 (3·9 - 9·9)
**Rwanda**	14	59	0	268	18 (13·1 - 21·0)	0 (0 - 3·2)	17·3 (12·4 - 20·0)
**Peru**	14	122	0	300	28·9 (23·5 - 33·2)	0 (0 - 1·3)	28·0 (22·3 - 32·2)
**90th percentile**							
**India**	1	0	37	366	0 (0 - 0)	97·4 (93·6 - 100)	9·2 (7·2 - 12·7)
**Guatemala**	27	20	2	340	5·6 (2·1 - 6·3)	6·9 (3·3 - 36·2)	5·7 (3·6 - 8·0)
**Rwanda**	22	51	0	268	16 (8·1 - 17·7)	0 (0 - 8·5)	15 (7·5 - 16·3)
**Peru**	31	105	0	300	25·9 (19·7 - 30·0)	0 (0 - 5·7)	24·1 (17·9 - 28·0)

e-Table 7. Misclassification rates for tachypnoea when using respiratory rate from Masimo Rad-97 only. In this table, **a** is the number of children with a RR ≥ 95th percentile and WHO upper thresholds (≥60 breaths/min for children 0-1 months, ≥50 breaths/min for children 2-11 months, ≥40 breaths/min for children 12-23 months); **b** is the number of children with a RR ≥ WHO upper threshold but below the 95th percentile; **c** is the number of children with a RR ≥ 95th percentile but below the WHO upper threshold; and **d** is the number of children with a RR below 95th percentile and WHO upper threshold. In this table, a = true positives, b = false positives, c = false negatives, and d = true negatives. Here, we define a false positive rate for tachypnoea (using the 95th percentile as the reference standard) as $\frac{b}{b+d}$, a false negative rate for tachypnoea as $\frac{c}{a+c}$, and the total misclassification rate for tachypnoea as $\frac{b+c}{a+b+c+d}$. We repeated the same calculations if we used 90th percentile of RR instead.

	**a =** Number of children with RR ≥ percentile and WHO upper threshold	**b =** Number of children with RR ≥ WHO upper threshold but < percentile	**c =** Number of children with RR ≥ percentile but < WHO upper threshold	**d =** Number of children with RR below percentile and WHO upper thresholds	% false positive for tachypnoea	% false negative for tachypnoea	% total misclassification
**95th percentile**							
**India**	1	0	22	381	0 (0 - 0·4)	95·7 (87·4 - 100)	5·4 (3·7 - 7·1)
**Guatemala**	38	9	14	328	2·7 (0·6 - 5·8)	26·9 (1·9 - 43·3)	5·9 (2·7 - 8·7)
**Rwanda**	31	42	0	268	13·5 (8·8 - 18·2)	0 (0 - 14·8)	12·3 (8·4 - 16·7)
**Peru**	32	104	0	300	25·7 (20·3 - 29·2)	0 (0 - 10·7)	23·9 (18·2 - 27·5)
**90th percentile**							
**India**	1	0	44	359	0 (0 - 0)	97·8 (92·3 - 100)	10·9 (7·8 - 12·2)
**Guatemala**	45	2	36	306	0·6 (0 - 2·3)	44·4 (27·0 - 60·3)	9·8 (5·9 - 13·7)
**Rwanda**	55	18	7	261	6·5 (2·3 - 11·9)	11·3 (2·5 - 20·4)	7·3 (4·7 - 11·2)
**Peru**	66	70	2	298	19 (12·9 - 23·3)	2·9 (0 - 15·8)	16·5 (11·5 - 20·9)

e-Table 8. Misclassification rates for hypoxaemia in children aged 2-11 months. In this table, **a** is the number of children with an SpO_2 below the 5th percentile and the WHO lower threshold (<90% at altitudes ≤2,500 m and <87% at altitudes above 2,500 m); **b** is the number of children with an SpO_2 below the WHO lower threshold but above or equal to 5th percentile; **c** is the number of children with an SpO_2 below the 5th percentile but above or equal to the WHO lower threshold; and **d** is the number of children with an SpO_2 above or equal to the 5th percentile and the WHO upper threshold. In this table, a = true positives, b = false positives, c = false negatives, and d = true negatives. Here, we define a false positive rate for hypoxaemia (using the 5th percentile as the reference standard) as $\frac{b}{b+d}$, a false negative rate for hypoxaemia as $\frac{c}{a+c}$, and the total misclassification rate for hypoxaemia as $\frac{b+c}{a+b+c+d}$. We repeated the same calculations if we used the 10h percentile of SpO_2 instead.

		**a =** Number of children with SpO_2 < percentile and WHO lower threshold	**b =** Number of children with an SpO_2 < WHO lower threshold but ≥ percentile	**c =** Number of children with SpO_2 < percentile but ≥ WHO lower threshold	**d =** Number of children with SpO_2 ≥ percentile and WHO upper threshold	% false positive for hypoxaemia (95% bootstrap CI)	% false negative for hypoxaemia (95% bootstrap CI)	% total misclassification (95% bootstrap CI)
**5th percentile**								
	India	0	0	7	119	0 (0 - 0)	100 (100 - 100)	5·6 (2·5 - 9·7)
	Guatemala	0	0	1	118	0 (0 - 0)	100 (100 - 100)	0·8 (0 - 5·2)
	Rwanda	0	0	3	123	0 (0 - 0)	100 (100 - 100)	2·4 (0 - 6·1)
	Peru	5	19	0	124	13·3 (7·4 - 18·3)	0 (0 - 0)	12·8 (7·0 - 17·6)
**10th percentile**								
	India	0	0	16	110	0 (0 - 0)	100 (100 - 100)	12·7 (5·1 - 15·6)
	Guatemala	0	0	8	111	0 (0 - 0)	100 (100 - 100)	6·7 (2·5 - 12·1)
	Rwanda	0	0	6	120	0 (0 - 0)	100 (100 - 100)	4·8 (0 - 11·2)
	Peru	13	11	0	124	8·1 (1·4 - 13·1)	0 (0 - 4·2)	7·4 (1·3 - 11·8)

e-Figure 1. Residual plot for the models using the Box-Cox Power Exponential distribution fitted to mean respiratory rate (panel A) and oxyhaemoglobin saturation (panel B). Worm plots of the residuals of the fitted models show that all points are close to the horizontal indicating that the Box-Cox Power Exponential distribution and regression models provide an appropriate fit to the data.

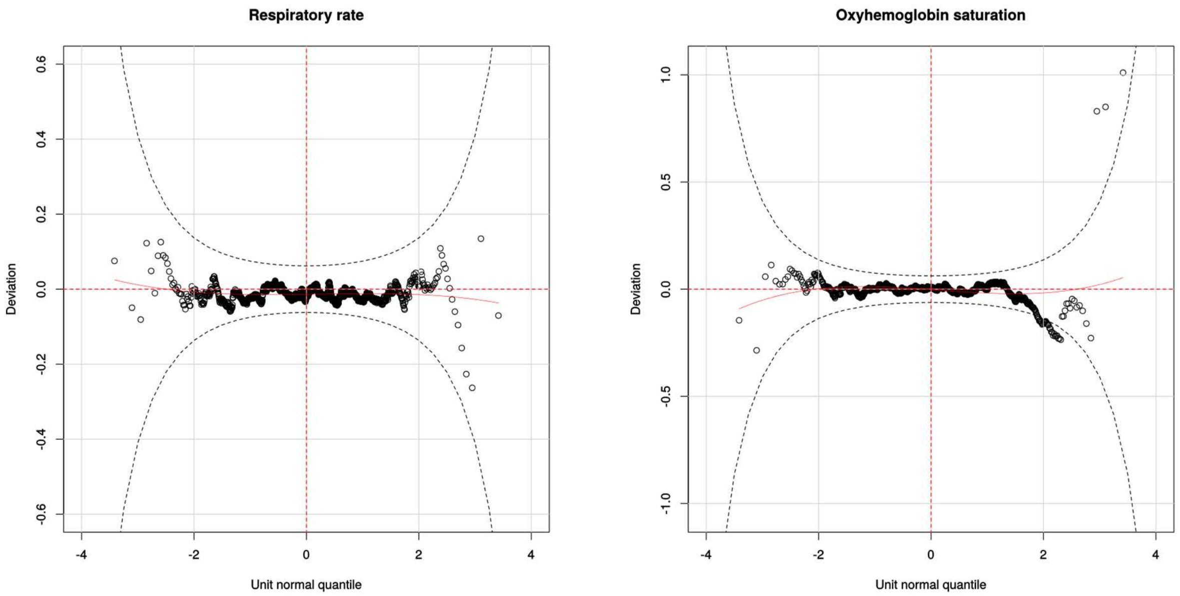

e-Figure 2. Hypothetical figure describing the process of classification in our analyses. Here we show hypothetical data of respiratory rate (breaths/minute) in the y-axis by age (months) in the x-axis. The blue step line represents the upper thresholds for respiratory rate defined by the World Health Organization (WHO), and the red line represents a hypothetical 95th age-specific smooth percentile. We divided the figure into four areas: children with a respiratory rate above or equal to the 95th percentile and WHO upper thresholds (a, in red); children with a respiratory rate above or equal to the WHO upper threshold but below the 95th percentile (b, in blue); children with a respiratory rate above or equal the 95th percentile but below the WHO upper threshold (c, in green); and, children with a respiratory rate below the 95th percentile and WHO upper thresholds (d, in black). Here, we define a false positive rate for tachypnoea (using the 95th percentile as the reference standard) as $\frac{b}{b+d}$, , a false negative rate for tachypnoea as $\frac{c}{a+c}$, and the total misclassification rate for tachypnoea as $\frac{b+c}{a+b+c+d}$.

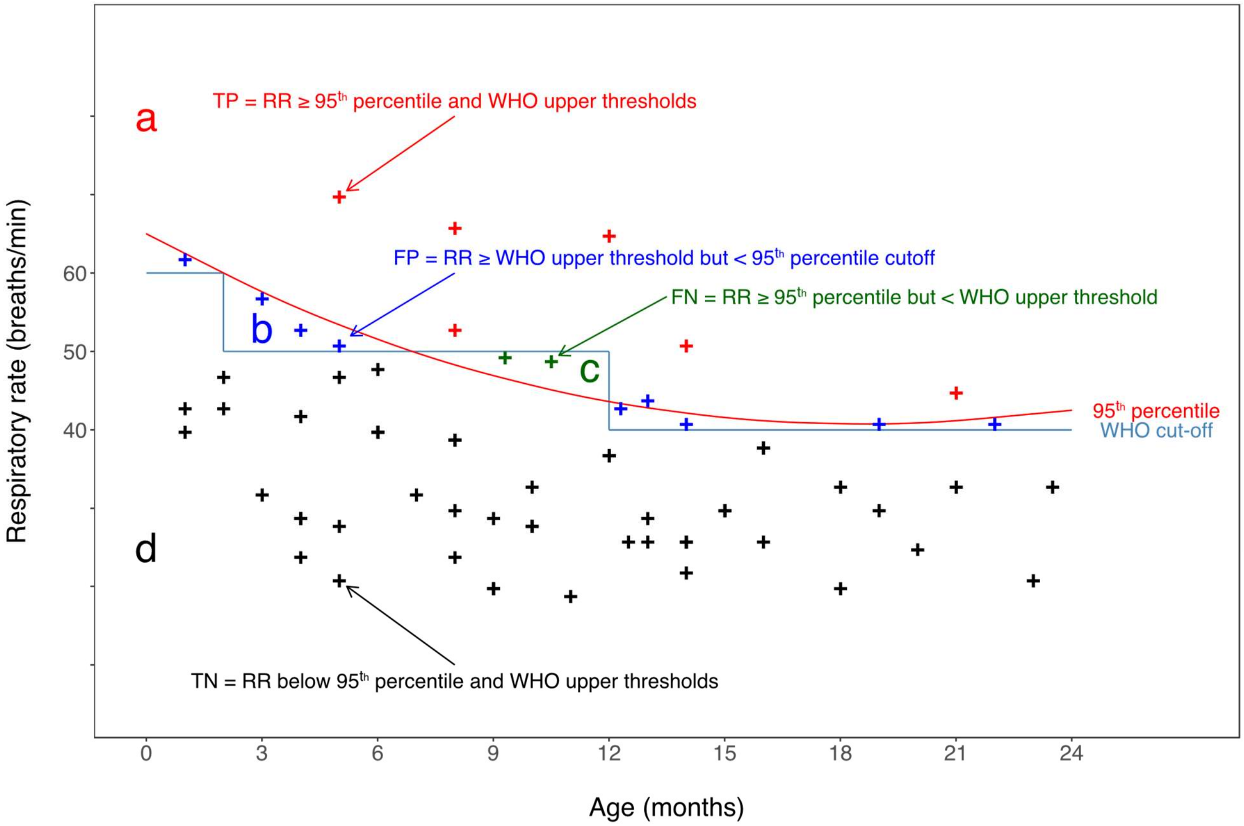

e-Figure 3. Flowchart of participants in the study.

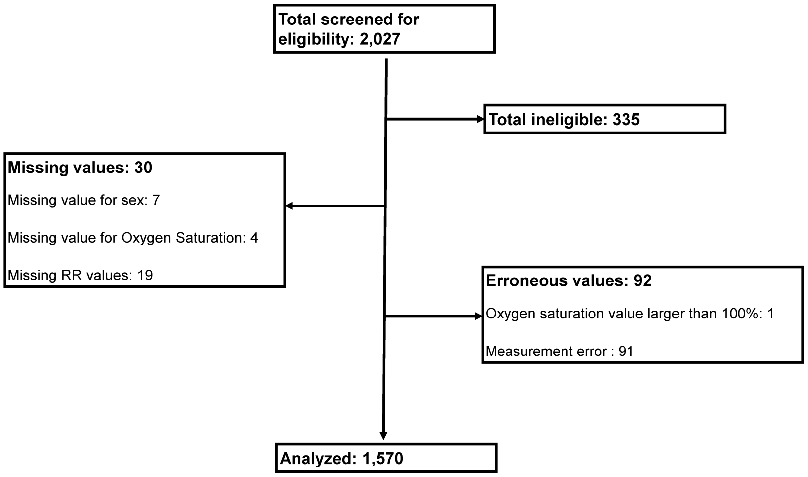

e-Figure 4. Age-specific smooth 95th percentiles for mean RR. We present estimated 95th percentiles obtained from a generalized additive regression model for location, shape, and scale by site. The observed data points for all settings are plotted in grey. Smooth lines represent 95th percentiles by age. The blue step line indicates the thresholds recommended by the World Health Organization for determining abnormal RR.

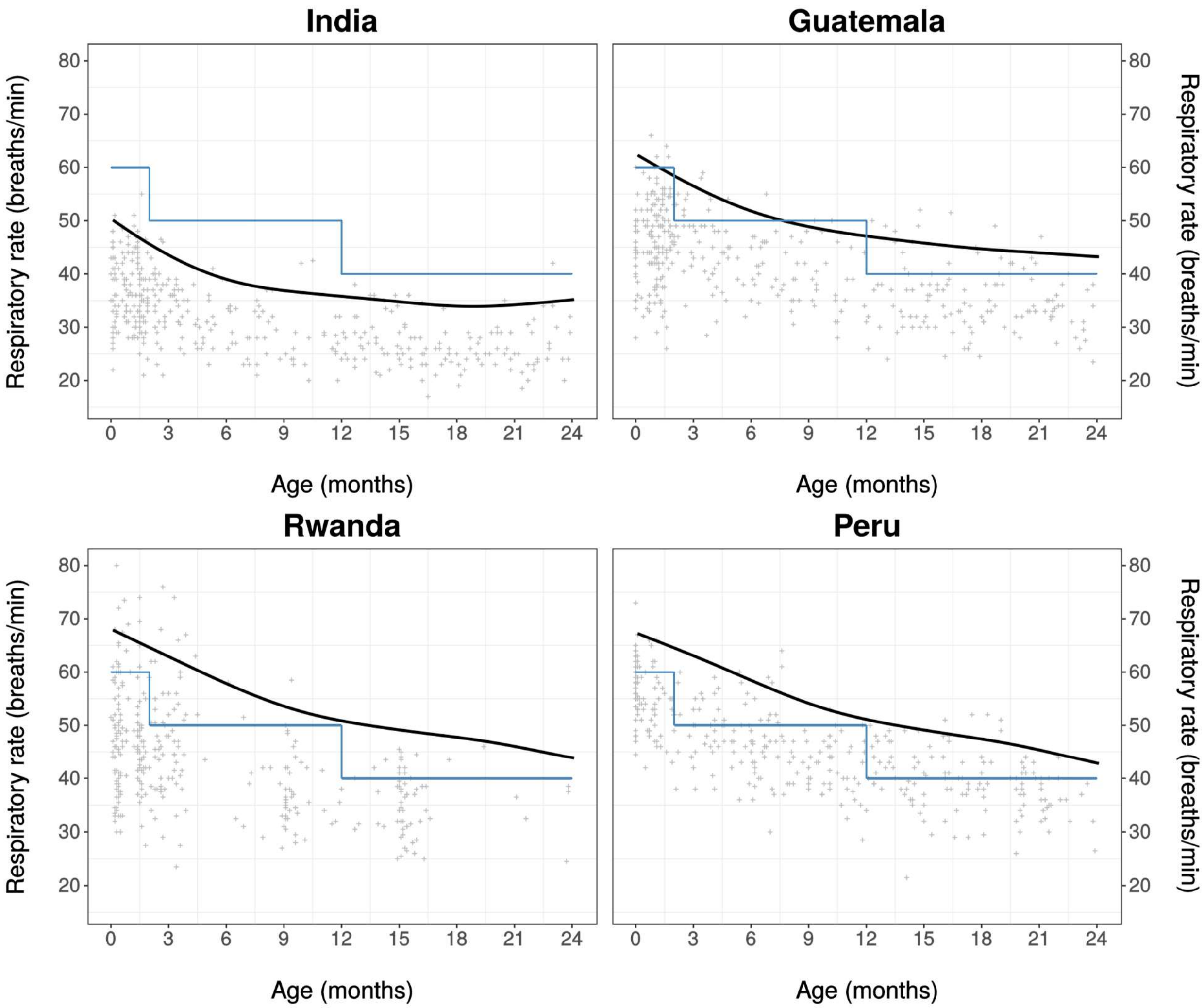

e-Figure 5. Age-specific smooth 5th percentiles for mean SpO_2. We present estimated 5th percentiles obtained from a generalized additive regression model for location, shape, and scale by site. The observed data points for all settings are plotted in grey. Smooth lines represent the 5th percentiles by age.

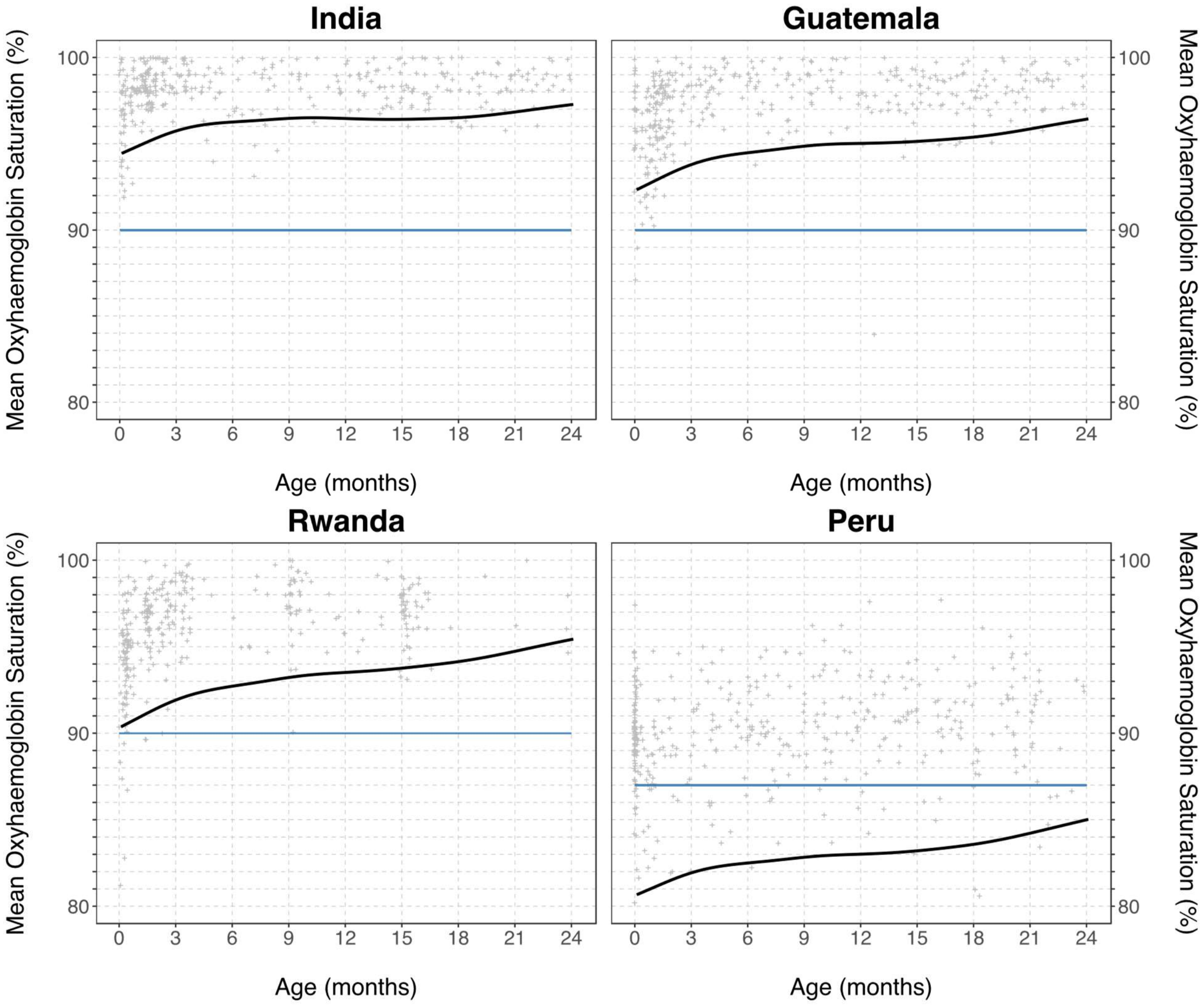

Supplement: Supplementary appendix [file mmc1.pdf]
